# *Announcement*: World Malaria Day — April 25, 2017

**DOI:** 10.15585/mmwr.mm6615a7

**Published:** 2017-04-21

World Malaria Day is commemorated each year on April 25, the date in 2000 when leaders of 44 African nations met in Abuja, Nigeria, and committed their countries to reducing the number of malaria-related deaths. Approximately 90% of all malaria deaths occur in Africa (*1*). During the last 15 years, donors have collectively supported the procurement and distribution of billions of insecticide-treated bed nets and courses of artemisinin-based combination therapy globally. These improvements in malaria control are estimated to have saved an estimated 6.8 million lives, mostly among children aged <5 years. From 2010 to 2015, the World Health Organization (WHO) reported that the estimated number of malaria deaths worldwide declined by approximately 60%, including a 69% decline among children aged <5 years (*1*). This year, as in 2016, the theme of World Malaria Day is “End Malaria for Good,” reflecting the increased interest in and commitment to eliminating malaria.

CDC provided support to the mid-20th century global public health push for malaria elimination, which resulted in eliminating local malaria transmission in the United States, much of Europe, nearly all of the Caribbean, and parts of the Middle East. Current malaria control initiatives led by WHO, including the Roll Back Malaria Partnership (http://www.rollbackmalaria.org/); the Global Fund to Fight AIDS, Tuberculosis and Malaria (https://www.theglobalfund.org/en/); and the U.S. President’s Malaria Initiative (https://www.pmi.gov/), working in partnership with countries with endemic malaria transmission, have contributed to important reductions in malaria incidence and deaths during the last 15 years. CDC conducts multidisciplinary research globally to increase knowledge about malaria and develop safe, effective interventions that can lead to the elimination and eventual eradication of malaria. CDC also provides evidence-based recommendations to protect Americans from malaria while living, working, and traveling in countries where malaria is endemic. Additional information about CDC's malaria activities is available at https://www.cdc.gov/malaria.

## References

[1] World Health Organization. Fact sheet: world malaria report 2016, December 13, 2016. Geneva, Switzerland: World Health Organization; 2016. http://www.who.int/malaria/media/world-malaria-report-2016/en/

